# *Yersinia pseudotuberculosis* Spatially Controls Activation and Misregulation of Host Cell Rac1

**DOI:** 10.1371/journal.ppat.0010016

**Published:** 2005-10-14

**Authors:** Ka-Wing Wong, Ralph R Isberg

**Affiliations:** 1 Department of Molecular Biology and Microbiology, Tufts University School of Medicine, Boston, Massachusetts, United States of America; 2 Howard Hughes Medical Institute, Tufts University School of Medicine, Boston, Massachusetts, United States of America; Yale School of Medicine, United States of America

## Abstract

*Yersinia pseudotuberculosis* binds host cells and modulates the mammalian Rac1 guanosine triphosphatase (GTPase) at two levels. Activation of Rac1 results from integrin receptor engagement, while misregulation is promoted by translocation of YopE and YopT proteins into target cells. Little is known regarding how these various factors interplay to control Rac1 dynamics. To investigate these competing processes, the localization of Rac1 activation was imaged microscopically using fluorescence resonance energy transfer. In the absence of translocated effectors, bacteria induced activation of the GTPase at the site of bacterial binding. In contrast, the entire cellular pool of Rac1 was inactivated shortly after translocation of YopE RhoGAP. Inactivation required membrane localization of Rac1. The translocated protease YopT had very different effects on Rac1. This protein, which removes the membrane localization site of Rac1, did not inactivate Rac1, but promoted entry of cleaved activated Rac1 molecules into the host cell nucleus, allowing Rac1 to localize with nuclear guanosine nucleotide exchange factors. As was true for YopE, membrane-associated Rac1 was the target for YopT, indicating that the two translocated effectors may compete for the same pool of target protein. Consistent with the observation that YopE inactivation requires membrane localization of Rac1, the presence of YopT in the cell interfered with the action of the YopE RhoGAP. As a result, interaction of target cells with a strain that produces both YopT and YopE resulted in two spatially distinct pools of Rac1: an inactive cytoplasmic pool and an activated nuclear pool. These studies demonstrate that competition between bacterial virulence factors for access to host substrates is controlled by the spatial arrangement of a target protein. In turn, the combined effects of translocated bacterial proteins are to generate pools of a single signaling molecule with distinct localization and activation states in a single cell.

## Synopsis

Many disease-causing bacteria transfer proteins into host cells, interfering with defense against infections. Bacteria often do this by manipulating host proteins that send signals. This study analyzes how one such bacterial pathogen manipulates the host signaling protein Rac1. The proteins YopT and YopE, which are made by several pathogens, including the agent of bubonic plague, had been presumed to inactivate Rac1. The authors show here that this model is too simple, and that pathogens are able to both inactivate and maintain activation of a host protein in a single cell. In this work, the pathogen divides up the Rac1 population into two pools, each with different potentials to send signals. One pool is found in the host cell cytoplasm and is unable to function properly. The other pool of Rac1 is sent into the nucleus, where it still sends an appropriate signal. Therefore, a bacterial pathogen is shown to allow signaling from one site in the host cell, while preventing it from occurring at a different site. Such locale-dependent events within single cells were not previously thought to play a role in microbial pathogenesis.

## Introduction

After ingestion by a host, *Yersinia pseudotuberculosis* gains a foothold in intestinal lymph nodes by moving across M cells into Peyer's patches [1]. As is true for the closely related *Y. enterocolitica,* entry into regional lymph nodes in the first few hours after infection requires the bacterial outer membrane protein invasin [2,3], which binds multiple β_1_ integrin receptors [1,4]. The preferential targeting of enteropathogenic *Yersinia* into M cells may be a consequence of specificity for these receptors, as no other cell type presents the appropriate integrin receptors on the intestinal lumen [5]. The importance of invasin for causing localized disease and colonizing within the intestine is well established [6], although the protein may be dispensable once bacteria establish infection in deep tissues [7,8].

Invasin-mediated uptake by cultured cells involves internalization of bacteria into membrane-bound compartments [9]. This adhesion event initiates actin rearrangements [4] controlled by the small guanosine triphosphatase (GTPase) Rac1 [10,11]. Rac1 is a member of the Rho GTPase family that controls a number of actin-dependent events, such as cell ruffling, motility, phagocytosis, and synapse formation [10–12]. Polyvalent engagement of integrin receptors by invasin results in activation of Rac1, with immediate loading of GTP into Rac1, although the site within the cell that harbors the activated Rac1 is not known [10]. The details that link integrin engagement to Rac1 activation are also unclear, although it has been argued that during integrin adhesion events, GTP loading produces a signal that releases cytosolic Rac1 from the protein Rho GDP-dissociation inhibitor (RhoGDI) [13]. Similarly unclear are the molecular details of how Rac1 coordinates the cytoskeletal rearrangements necessary for invasin-mediated uptake. Overproduction and localization studies argue that actin dynamics promoted by Arp2/3 and the lipid phosphoinositol-4,5-phosphate, as well as the small GTPase Arf6, are players downstream from Rac1 during uptake [14].

*Yersinia* species also target Rac1 for inactivation by several translocated effector proteins delivered by a type III secretion system, so the dynamics of Rac1 nucleotide loading must result from interplay between these proteins [15]. Two of these effectors, YopE and YopT, interfere with the function of multiple Rho family members through distinct mechanisms. YopE is a GTPase-activating protein for Rho family members (RhoGAP) [16,17]. The primary consequence of the RhoGAP activity of YopE is to interfere with a wide variety of phagocytic events, including invasin-mediated uptake. *Pseudomonas aeruginosa* ExoS and *Samonella typhimurium* SptP RhoGAP proteins have been shown to have similar functions [18,19]. Another translocated effector that inhibits invasin-mediated uptake is YopT, which is a cysteine protease that cleaves upstream of the prenylated cysteine within the CAAX motif at the C terminus of Rho family members Cdc42, Rac1, and RhoA [20]. Although purified YopT protein from *Y. pestis* does not appear to show specificity for individual Rho family members, YopT proteins from different species of *Yersinia* may not behave identically. For instance, the *Y. enterocolitica* protein targets only RhoA after its translocation into macrophages [21]. The substrate specificity of YopT in *Y. pseudotuberculosis-*infected cells is not known. Also unknown are the consequences of YopT cleavage on the activation state of Rho family members [22].

Enteropathogenic *Yersinia* species transmit potentially conflicting signals to Rho GTPases that may accommodate specific requirements during establishment of disease. To study these signals, we analyzed the response of Rac1 to adhesion by *Y. pseudotuberculosis*. We found that intracellular localization of Rac1 controls the ability of the bacterium to manipulate activation of this regulatory molecule.

## Results

### Use of Fluorescent Resonance Energy Transfer Measurements to Monitor Rac1 Activation

Several strategies based on fluorescence resonance energy transfer (FRET) have been described to visualize Rac1 activation [23–25]. To determine the consequences of *Y. pseudotuberculosis* infection on Rac1 activation in host cells, we developed a strategy similar to that reported previously [23], in which the loading of GTP into Rac1 was localized to specific sites in the mammalian cell using fluorescence microscopy. Activated (GTP-loaded) Rac1 can be distinguished from the rest of the Rac1 pool in the cell by its ability to bind the effector domain of PAK1 (residues 67–118 from p21-associated kinase, called p21-binding domain [PBD]). In cells transfected with CFP-tagged Rac1, binding by the GTP-loaded form of Rac1 to YFP-tagged PBD was measured by intermolecular fluorescent resonance energy transfer from the CFP moiety to the YFP moiety after 440 nm excitation (Figure 1A). As YFP and CFP can dimerize and generate a FRET signal, monomeric derivatives of these proteins were used (called mCFP and mYFP) [26]. Throughout the work, sensitized FRET is defined as the total amount of FRET in a defined region of interest, using the appropriate corrections from images captured by fluorescence microscopy (Materials and Methods). Normalized FRET is the ratio of sensitized FRET divided by the total amount of a fluorophore (usually mCFP-Rac1) in a region of interest (Materials and Methods). Except where noted, we excluded the nuclear pool of Rac1 in the analysis of mCFP-Rac1 activation.

**Figure 1:**
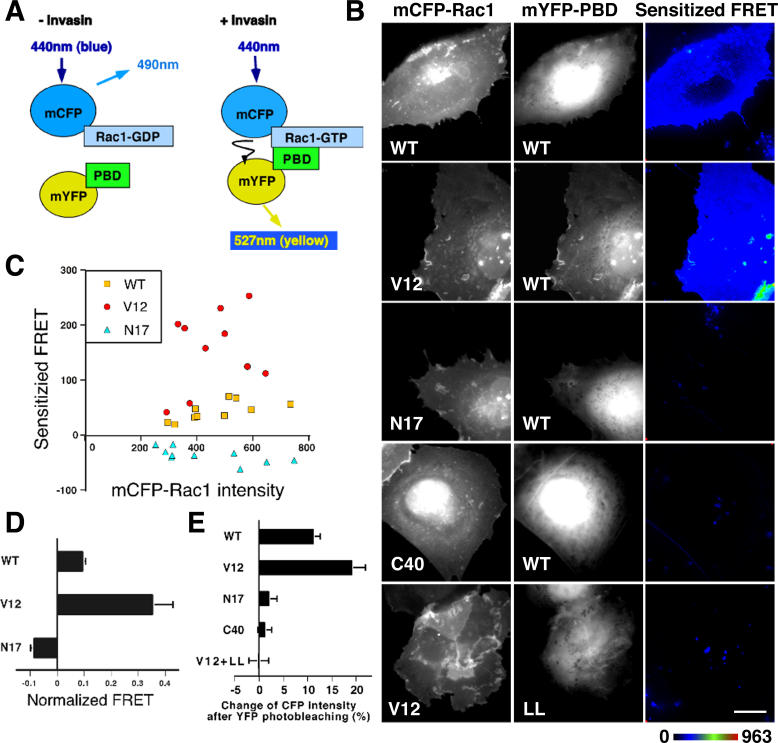
Activation of Rac1 in Single Cells as Demonstrated by FRET (A) Schematic representation of intermolecular FRET from Rac1-GTP to PBD. In the absence of GTP loading, mCFP-Rac1 is unable to bind mYFP-PBD. Upon GTP loading (activation) of Rac1, mCFP-Rac1-GTP is able to bind to the downstream effector construction mYFP-PBD. This allows the emission from mCFP to excite mYFP, resulting in emission at 527 nm. (B) Display of fluorescence emission and sensitized FRET in individual transfectants. COS1 cells cotransfected with various mCFP-Rac1 derivatives and either mYFP-PBD or mYFP-PBD(LL) were subjected to FRET analysis, and sensitized FRET was calculated (Materials and Methods). Displayed are the channels for CFP and YFP emission as well as the relative Rac1 activation throughout the cell as determined by sensitized FRET. The color-scale bar represents the amount of Rac1 activation displayed as relative pixel intensity in noted area of cell (Materials and Methods). White bar represents 10 μm. (C) FRET is dependent on coexpression of active mCFP-Rac1 and mYFP-PBD. FRET from ten ROIs, which represented two cytoplasmic regions from each of five cells, were plotted as a function of the intensity of the mCFP-Rac1 donor. (D) Activation of Rac1 increases normalized levels of FRET. Data from (C) were normalized to amount of mCFP-Rac1 in each region of interest and displayed. Displayed are means ± standard errors of the mean (SEMs) (E) Enhanced emission of the mCFP-Rac1 donor resulting from acceptor photobleaching requires Rac1 activation. The acceptor mYFP-PBD was selectively photobleached for 2 min, and amount of mCFP-Rac1 donor emission was compared before and after irradiation (Materials and Methods). The percentage increase in emission of after irradiation is displayed. To determine emission enhancement, ten ROIs were analyzed. Displayed are means ± SEMs.

To determine if GTP loading was the specific readout of this system, a series of mutant Rac1 and PBD derivatives were transfected into COS1 cells in the absence of incubation with *Y. pseudotuberculosis* (Table 1). The constitutively active mCFP-Rac1V12 (GTP-bound, Table 1) generated substantial FRET, as predicted for a protein that does not require external activation (Figure 1B and 1C). GTP loading of Rac1 was required to detect a signal, because no sensitized FRET was observed in cells cotransfected with the dominant negative mCFP-Rac1N17 and mYFP-PBD (Figure 1B). The intensity of sensitized FRET observed was a function of the concentration of mCFP-Rac1 (mCFP-Rac1 intensity; Figure 1C) and mYFP-PBD (unpublished data) in individual transfectants. Cells cotransfected with mCFP-Rac1 wild-type (WT) and mYFP-PBD produced FRET at an expected lower efficiency than Rac1V12, based on the levels of FRET normalized to the concentration of Rac1 (Figure 1C and 1D), as only a portion of the Rac1 pool would be predicted to be GTP loaded in these cells. Consistent with the FRET observed being due to direct binding of PBD to Rac1 rather than simple colocalization of the two proteins, we found that no FRET was observed when the PBD binding-defective mCFP-Rac1C40 (Table 1) was cotransfected with mYFP-PBD (Figure 1B). Furthermore, no FRET was observed when a mutant mYFP-PBD defective in binding to activated Rac1 (PBD-LL; Table 1) was analyzed (Figure 1B). Thus, sensitized FRET measurements monitored the activation status of Rac1 as well as the concentration of Rac1 and its effector PBD in a single cell, and the controls are consistent with FRET resulting from direct binding of Rac1 to effector.

**Table 1:**
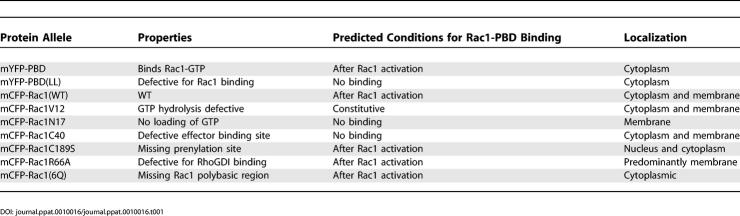
Predicted Properties of Fluorescent Proteins Used in this Study

To give further support to the hypothesis that FRET was due to binding of Rac1-GTP to PBD, photobleaching was performed (Figure 1E). When activated mCFP-Rac1 binds mYFP-PBD, the mYFP acceptor absorbs a fraction of the fluorescence emission from the mCFP donor, resulting in lost mCFP emission. The lost mCFP emission can be restored by destroying the mYFP fluorophore via photobleaching (Materials and Methods) [27]. As expected, cells cotransfected with mCFP-Rac1(WT) and mYFP-PBD resulted in a 10% increase in mCFP emission after photobleaching of mYFP-PBD (Figure 1E). Photobleaching of the mYFP-PBD acceptor resulted in an even higher enhancement of mCFP-Rac1V12 emission (16%). Identical photobleaching treatments, however, had almost no effect on the emission intensities of the nonbinding mCFP-Rac1N17 or mCFP-Rac1C40 mutants (Figure 1E). Similarly, no enhancement of mCFP-Rac1 emission was observed after photobleaching of mYFP-PBD(LL), consistent with the fact that this protein is defective for interaction with activated Rac1. Therefore, by two different criteria we can detect specific binding of mYFP-PBD to Rac1-GTP.

### GTP-Loaded Rac1 Is Recruited to Nascent Phagosomes

Rac1 is activated after engagement of integrin receptors by *Y. pseudotuberculosis* [10]. This could occur as either global activation of the entire Rac1 pool or localized changes in Rac1 conformation at regions proximal to the nascent phagosome. To distinguish between these possibilities, FRET analysis was performed after 20 min incubation of *Y. pseudotuberculosis* with COS1 cells. The *Y. pseudotuberculosis* strain used for these infections lacked the *Yersinia* virulence plasmid and was grown under conditions in which the invasin protein was the only adhesin expressed, so that all effects observed were a consequence of invasin engagement of receptors. When fixed samples were stained to identify partially internalized bacteria (Figure 2; see Materials and Methods), the mCFP-Rac1(WT) recruited around nascent phagosomes showed concentrated levels of activation (Sensitized FRET, Figure 2A; WT, Figure 2E). This could result from either simple recruitment of mCFP-Rac1 or the presence of a pool of mCFP-Rac1 localized around the phagosomes that is more highly activated than pools found elsewhere in the cell. To distinguish between these possibilities, the amount of activated mCFP-Rac1 was normalized to the concentration of mCFP-Rac1 at specific points within the cell. When this was taken into account, it was clear that the mCFP-Rac1 associated with both the nascent phagosomes and the cell periphery was in a more highly active state then pools found elsewhere in the cell (Normalized FRET, Figure 2A; WT, Figure 2F; *p* < 0.006). In contrast, although active mCFP-Rac1V12 was concentrated around nascent phagosomes (Sensitized FRET, Figure 2B; V12, Figure 2E), no enhanced activity could be observed around entering bacteria relative to other sites in the cell, as there was active mCFP-Rac1V12 throughout the cell (Normalized FRET, Figure 2B; V12, Figure 2F). The mCFP-Rac1N17 derivative, which fails to load GTP, showed no enhanced activation (Figure 2C), while the mCFP-Rac1C40 mutant that fails to bind PBD also produced no FRET with mYFP-PBD (Figure 2D). As mCFP-Rac1C40 is localized around nascent phagosomes, this demonstrates that mere concentration of mCFP-Rac1 around the phagosome is not sufficient to generate a FRET signal. These data indicate that invasin binding to host cells results in a clear concentration of Rac1 in the activated form at the nascent phagosome.

**Figure 2:**
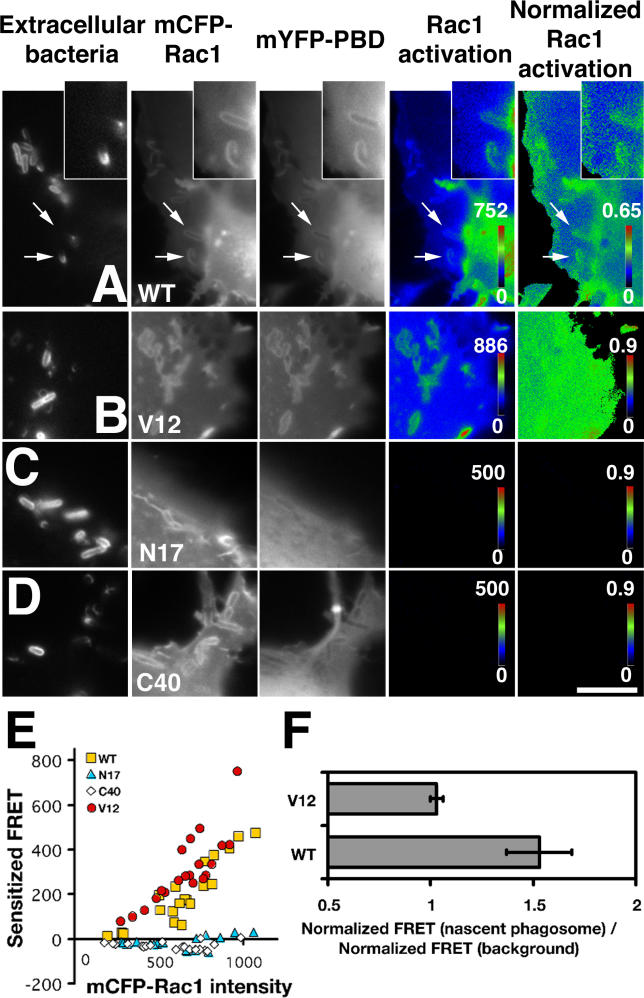
Localized Activation of Rac1 on the Phagosomal Membrane during *Y. pseudotuberculosis* Uptake (A–D) Transfected COS1 cells were incubated with *Y. pseudotuberculosis* (YPIII[P^−^]) for 20 min, fixed, and then immunostained with anti-*Y. pseudotuberculosis* to detect partially internalized bacteria (Extracellular bacteria; see Materials and Methods). Rac1 activation was measured by sensitized FRET, and normalization (Normalized FRET) was performed by determining the amount of FRET at each pixel relative to the mCFP-Rac1 intensity at that pixel (Materials and Methods). Color-gradient scales, as described in Figure 1, were used to represent the intensity of signal for Rac1 activation (Sensitized FRET) as well as for normalized Rac1 activation (Normalized FRET). Note the scales are not identical in each window. Arrows indicate nascent phagosomes, as described (Materials and Methods), and these are simultaneously displayed in insets. Shown are cotransfection of: mCFP-Rac1(WT) and mYFP-PBD (A), constitutively active mCFP-Rac1V12 with mYFP-PBD (B), dominant negative mCFP-Rac1N17 with mYFP-PBD (C), and effector binding-defective mCFP-Rac1C40 with mYFP-PBD (D). White bar represents 10 μm. (E) FRET in response to *Y. pseudotuberculosis* adhesion is dependent on coexpression of active mCFP-Rac1 and mYFP-PBD. Sensitized FRET (Rac1 activation) at regions surrounding nascent phagosomes was plotted as a function of the intensity of the mCFP-Rac1 donor in the cell. (F) The pool of Rac1 surrounding incoming bacteria is preferentially activated. The intensity of FRET that surrounded nascent phagosomes was compared to that found in nearby cytoplasmic areas, normalized against the concentration of mCFP-Rac1 found in each area, and then used to calculate the ratio of normalized Rac1 activity of nascent phagosome to that of background (Materials and Methods). Data from 20 nascent phagosomes in cells expressing mYFP-PBD along with either mCFP-Rac1(WT) or mCFP-Rac1V12 were displayed as mean ± SEM (*p* = 0.006).

### Selective Inactivation of Membrane-Targeted Rac1 by YopE

The ability to localize activated Rac1 to specific sites within target cells facilitated spatial analysis of the targeting of Rac1 by *Yersinia* translocated effectors, allowing investigation of the consequences of YopE RhoGAP activity (Figure 3) [16,17]. Using a strain that expresses invasin and YopE (YP17/pYopE, Materials and Methods) but lacks the effectors YopT and the tyrosine phosphatase YopH, there was little GTP-loaded mCFP-Rac1 in target cells (Figure 3A and 3C). To determine whether the cytosolic or membrane pools of Rac1 were inactivated, two Rac1 mutants were analyzed that have altered localization properties. The Rac1R66A mutant (Table 1) does not bind to RhoGDI, and because RhoGDI is required to extract Rac1 from the membrane, the mutant protein localizes in the membrane [28]. The second mutant, Rac1C189S, does not undergo prenylation of the cysteine required for membrane localization, and remains soluble. When regions of interest (ROIs) within the cytoplasm of cells having bound *Y. pseudotuberculosis* were analyzed, it was clear that cytosolic mCFP-Rac1C189S remained active after YopE translocation (Figure 3B and 3C). The entire pool of the membrane-localized mCFP-Rac1R66A, in contrast, appeared to be inactivated (Figure 3B and 3C). Therefore, although YopE rapidly inactivates Rac1, it does so by selectively targeting membrane-associated Rac1. Presumably, the inactivation of mCFP-Rac1(WT) was due to YopE inactivation of membrane-associated protein, followed by translocation of any soluble pools of GTP-loaded mCFP-Rac1(WT) to the plasma membrane, and then further cycles of inactivation.

**Figure 3:**
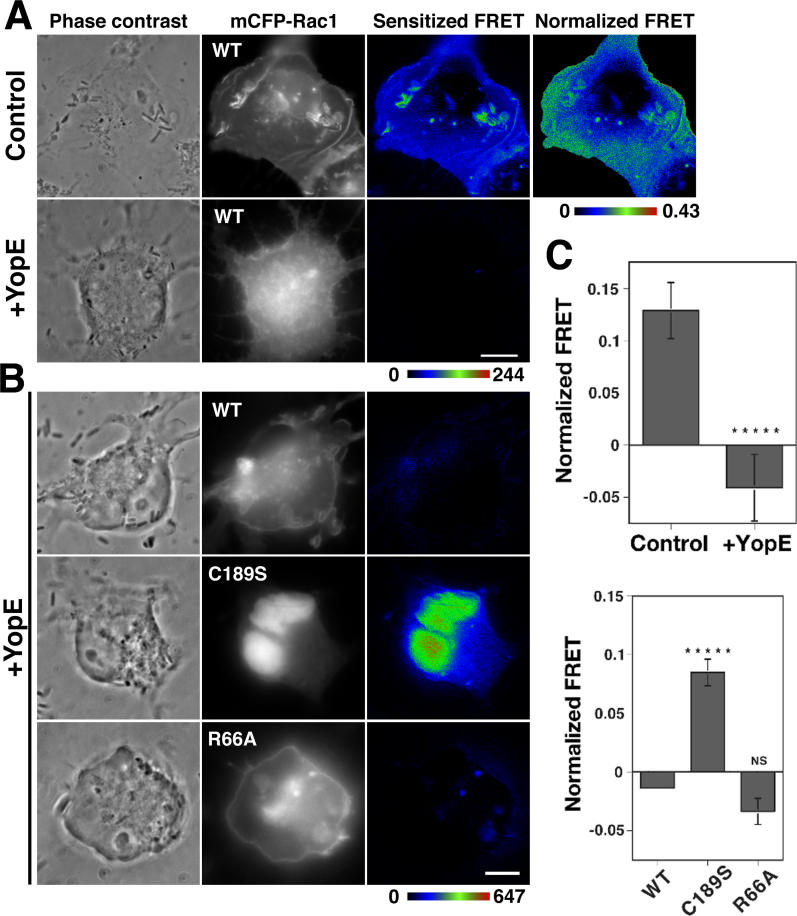
YopE Selectively Inactivates Membrane-Associated Rac1. COS1 cells cotransfected with plasmids expressing mYFP-PBD and noted plasmids were challenged at 37 °C for 30 min with *Y. pseudotuberculosis* YP17*(yopH ^−^, yopT ^−^, yopE ^−^)* (noted as “Control” in [A] and [B]) or YP17/pYopE (noted as “+YopE” in [A–C]). Bacteria were grown at 37 °C for 1 h to induce expression of the type III secretion system prior to infection (Materials and Methods). The C189S mutation blocks plasma membrane localization of Rac1, whereas R66A prevents extraction of Rac1 by RhoGDI from plasma membrane into cytosol. (A and B) Displayed are images of typical cells showing phase contrast, mCFP fluorescence and color-scaled sensitized FRET or normalized (Rac1 activation) for YP17 (A) or Yp17/pYopE (A and B) infected cells (Materials and Methods). White bar represents 10 μm. (C) Cytoplasmic Rac1 is not a YopE target. Normalized FRET was determined (Materials and Methods) by imaging cytoplasm of eight infected cells (Materials and Methods). Data for mCFP-Rac1 activation was normalized against mCFP-Rac1 intensity (normalized Rac1 activation) and displayed as mean ± SEM. *****, *p* < 5 × 10^−6^ between control cells and YopE-treated cells or between Rac1(WT) and Rac1C189S-transfected cells. NS, no significant difference between Rac1(WT)-transfected cells and Rac1R66A-transfected cells.

### The *Y. pseudotuberculosis* YopT Removes Multiple Rho Family Members from Nascent Phagosomes

We next wished to analyze the interplay between YopE and another translocated effector that targets Rac1 in vitro. Both YopE and YopT inhibit uptake of enteropathogenic *Yersinia* species (unpublished data) [17], although it is not clear that YopT targets Rac1 in intact cells. It has been previously reported that the *Y. enterocolitica* YopT cysteine protease selectively releases RhoA from the plasma membrane of target cells, inhibiting bacterial uptake [21]. This conflicts with analysis of *Y. pseudotuberculosis* indicating that cells having inactivated RhoA support invasin-dependent internalization, so presumably *Y. pseudotuberculosis* YopT must target another Rho family member [17].

To investigate the possibility that *Y. pseudotuberculosis* YopT also targeted Rac1 and Cdc42, we examined localization of Rho family members about the nascent phagosome (Figure 4). The three Rho family members RhoA, Cdc42, and Rac1 were recruited to nascent phagosomes in a strain lacking YopT, YopH, and YopE (YP17; Figure 4A and 4C). In contrast, all three Rho family members were depleted from partially formed phagosomes after 30 min of contact with the identical *Y. pseudotuberculosis* strain expressing YopT (Figure 4B and 4D). The percentage of adherent bacteria associated with each small GTPase in the presence of YopT was as low as that observed with Rac1C189S, which lacks a geranylgeranyl moiety to localize it on plasma membrane. In contrast, Arf6 localization [14,29] was not affected by YopT treatment, showing that the phagosomal membrane was not disrupted by YopT (Figure 4B and 4D). Therefore, at least in the case of *Y. pseudotuberculosis,* there does not appear to be striking substrate specificity for various Rho family members.

**Figure 4:**
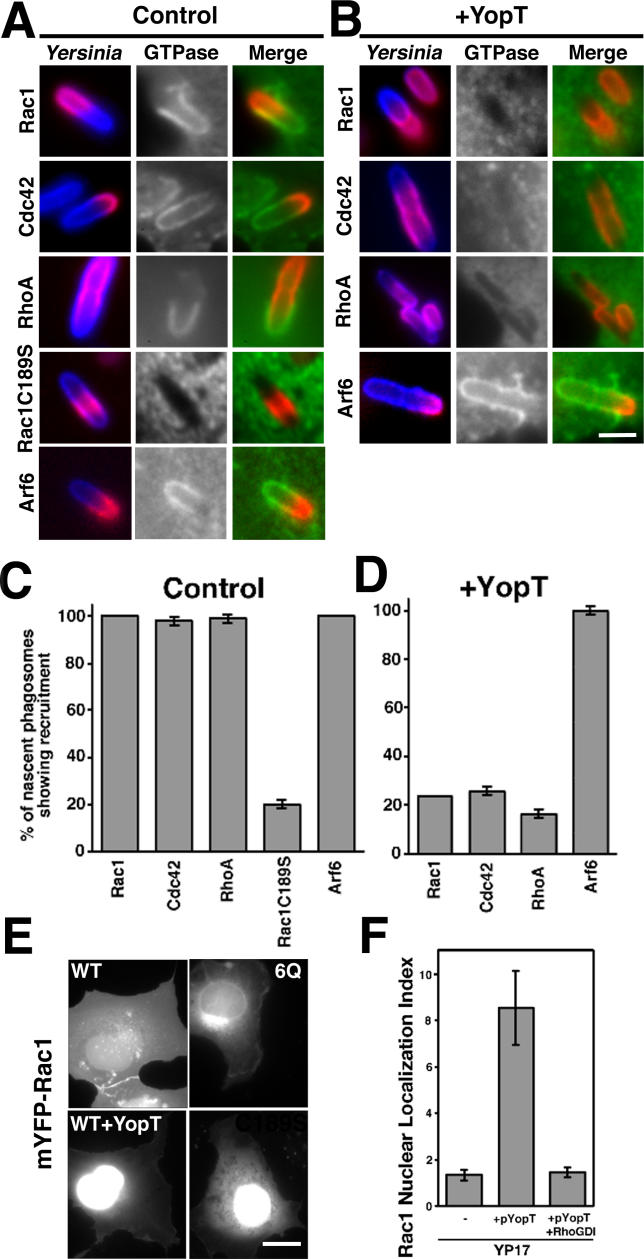
*Y. pseudotuberculosis* YopT Removes Multiple Rho Family Members from Nascent Phagosomes (A–D) COS1 cells transfected with plasmids encoding T7-Rac1, T7-Rac1C189S, myc-Cdc42, myc-RhoA, or HA-Arf6 were incubated at 37 °C for 30 min with *Y. pseudotuberculosis* YP17 (A and C) or YP17/pYopT (B and D) grown under described conditions (Materials and Methods). (A and B) To identify nascent phagosomes, bacteria were probed as described (Materials and Methods), and small GTPases were identified by immunoprobing against appropriate tags. Pink identifies the extracellular region of a bacterium and blue represents the internalized region. Green indicates the staining for small GTPases. (C and D) Quantifications of the percentage (mean ± SEM in triplicate) of partially internalized bacteria that stained positively for Rac1 (*n* = 33), Cdc42 (*n* = 33), RhoA (*n* = 33), Rac1C189S (*n* = 33), or Arf6-HA (*n* = 11). (E) Representative localization of uninfected cells expressing mYFP-Rac1(WT), mYFP-Rac1C189S, or mYFP-Rac16Q, as well as a mYFP-Rac1(WT) transfectant challenged with YP17/pYopT for 30 min (WT; +YopT). White bar represents 1 μm. (F) The presence of excess RhoGDI interferes with the activity of YopT. *Y. pseudotuberculosis* YP17/pYopT (+pYopT) was introduced for 30 min onto COS1 cells or transfectants overexpressing RhoGDI. Nuclear localization of Rac1 (defined as the ratio of the mean nuclear Rac1 intensity relative to the mean cytoplasmic Rac1 intensity) was determined in each case.

Rac1 has a strong nuclear localization signal in the polybasic domain located immediately upstream of the CAAX prenylation motif [22]. YopT-promoted cleavage should release the prenylation motif and liberate a truncated Rho family member that contains the intact nuclear localization [20]. After 30 min of YopT translocation from adherent *Y. pseudotuberculosis,* there was intense nuclear accumulation of Rac1 (69% of cells showed nuclear localization, *n* = 36; Figure 4E), which was similar in intensity to that observed for the Rac1C189S mutant (Figure 4E). This contrasts with the control incubation with bacteria that fail to express YopT (7.7% of cells showed nuclear localization, *n* = 39). Consistent with the importance of the polybasic region for this localization result, we observed no nuclear accumulation by the Rac16Q mutant (Table 1) in which the polybasic region was disrupted by replacement with six Gln residues (Figure 4E). These results indicate that removal of the prenylated cysteine by YopT induces Rac1 nuclear localization.

As observed for YopE, membrane-associated Rac1 was the target of YopT. Bacterial translocation of YopT failed to result in nuclear accumulation of the cytoplasmically localized Rac16Q (unpublished data). Furthermore, if Rac1 were localized in a cytoplasmic pool as a result of overproduction of RhoGDI, then YopT-promoted nuclear accumulation of Rac1 was blocked (Figure 4F). Therefore, both YopE and YopT targeted the identical membrane-associated fraction of Rac1, and potentially can compete for the same pool of Rac1.

### YopT Cleavage Does Not Block Rac1 Activation

Based on the biochemical properties of the *Yersinia* effectors, YopE disrupts function by preventing Rac1 activation, whereas YopT simply mislocalizes Rac1. It is possible, however, that the altered localization of Rac1 that results from YopT proteolysis also interferes with GTP loading. For this reason, we examined whether Rac1 mislocalization results in its inactivation, using the *Y. pseudotuberculosis* strain that expresses YopT as the only known translocated effector that alters Rac1 function. After 30 min of incubation with YP17/pYopT, transfected cells exhibited intense nuclear accumulation of mCFP-Rac1, indicating that most of the Rac1 was cleaved (Figure 5A). Despite this altered localization, there were high levels of GTP-loaded Rac1 (Figure 5B), which may have been activated by exchange factors known to localize in the nucleus, such as SmgGDS and Dock180 [22,30]. The highly concentrated mCFP-Rac1 in the nuclear region and the less highly concentrated pool in peripheral regions of the cell had similar levels of activation when normalized to the amount of mCFP-Rac1 in these regions (Figure 5A and unpublished data). The action of translocated YopT on Rac1(WT) mimicked the behavior of Rac1C189S in uninfected cells, as this derivative had similar activation levels compared to transfectants harboring mCFP-Rac1(WT) (Figure 5C–5E). Therefore, prenylation of Rac1 was not required for GTP-loading and effector binding of Rac1 in this system. In addition, in the absence of YopE, the primary role of YopT is to disrupt the localization of Rac1 without affecting its activation.

**Figure 5:**
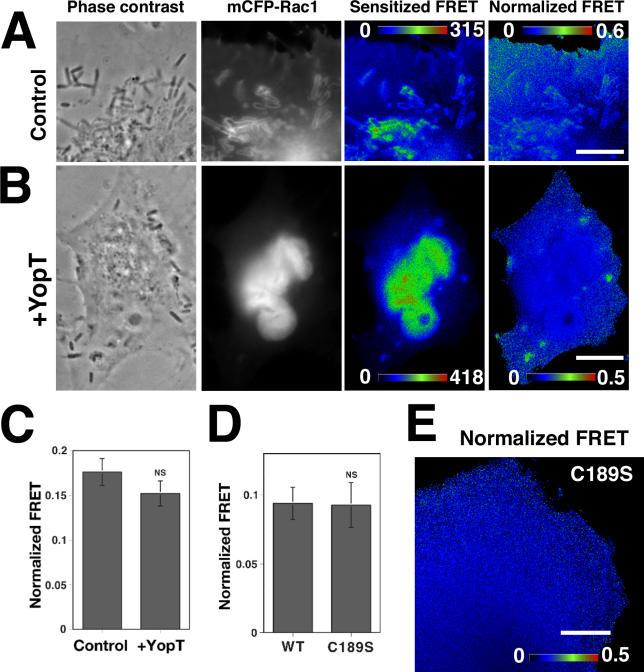
YopT Cleavage Does Not Prevent Rac1 Activation COS1 cells were cotransfected with plasmids expressing mYFP-PBD and either mCFP-Rac1 or mCFP-Rac1C189S followed by exposure to *Y. pseudotuberculosis* for 30 min (Materials and Methods) and FRET analysis in cytoplasmic ROIs. (A–C) Cells expressing mCFP-Rac1(WT) challenged with either YP17 (control) (A) or mCFP-Rac1(WT) challenged with YP17/pYopT (+YopT) (B). Rac1 activation is maintained in the presence of YopT cleavage (C). Data for normalized Rac1 activity from eight cells described in (A) and (B) were quantitated as mean ± SEM. (D) Cytoplasmic localization of Rac1 does not affect activation. FRET was analyzed in ROIs from cytoplasmic regions of uninfected cells transfected with mCFP-Rac1(WT) or mCFP-Rac1C189S and normalized versus amount of mCFP observed in each cell. NS, no significant difference between control and experimental. (E) Example of normalized FRET observed in uninfected cell expressing mCFP-Rac1C189S. White bar represents 10 μm.

### The Combined Action of YopE and YopT Generate Two Different Pools of Rac1

The above results indicate that the nonprenylated form of Rac1 is a poor substrate for YopE. Therefore, the removal of prenylation by YopT should protect a pool of Rac1 from inactivation by YopE, and that pool should move into the nucleus. To test this prediction, a strain expressing both YopE and YopT was used to challenge COS1 cells, and levels of Rac1 activation were compared to the parental strain lacking YopT (Figure 6). As the YPIII strain does not encode YopT, a plasmid expressing YopT was introduced into the strain (YP15/pYopT). YopT clearly interfered with the action of YopE (Figure 6A), with the level of cytoplasmic FRET approaching that observed in cells incubated with bacteria lacking YopE (Figure 6B), and it caused accumulation of activated Rac1 in the nucleus (Figure 6C). It appeared, however, that persistent activation of Rac1 under these conditions was probably due to overexpressed YopT blocking YopE translocation into COS1 cells. When translocation was analyzed using a standard detergent solubilization strategy [31], the strain expressing both proteins (YP15/pYopT; Figure 6D) showed barely detectable YopE translocation, with levels only slightly above those in strains lacking the type III secretion system (yscU, Figure 6D).

**Figure 6:**
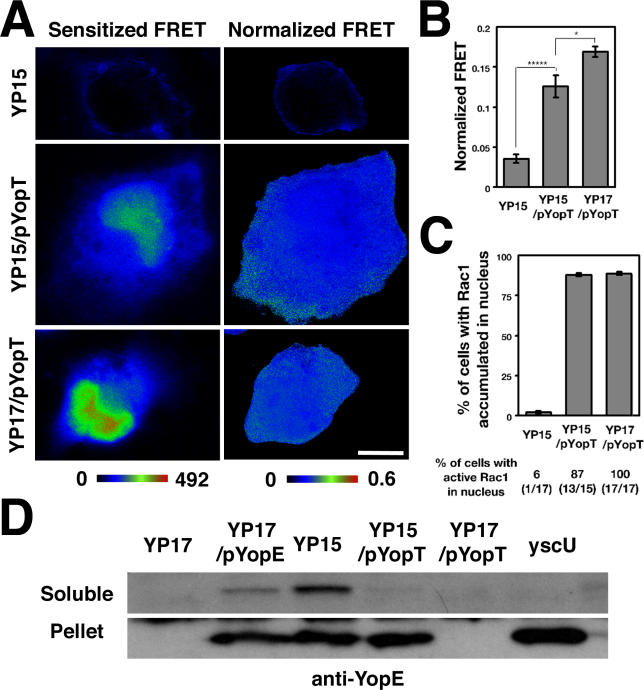
Overexpression of YopT Interferes with YopE-Mediated Inactivation of Rac1 by Blocking YopE Translocation COS1 cells were cotransfected with plasmids expressing mYFP-PBD and mCFP-Rac1, challenged for 30 min with *Y. pseudotuberculosis* strains YP15 *(yopE ^+^),* YP15/pYopT *(yopE ^+^yopT ^+^)* or YP17/pYopT *(yopE ^−^yopT ^+^)* and processed for FRET analysis as in Figure 5 (Materials and Methods). (A) Typical FRET images of cells incubated with denoted strains. (B) The presence of pYopT interferes with the ability of YopE to inactivate cytoplasmically localized Rac1. Data represent normalized FRET, using ROIs within the cytoplasms of ten cells. ***** *p* < 5 × 10^−5^; * *p* = 0.01. Normalized FRET was determined as described (Materials and Methods). (C) Plasmid-expressed YopT promotes translocation of active Rac1 into the nucleus in the presence of YopE. Nuclear accumulation of Rac1 was determined by presence of mCFP-Rac1 fluorescence (bar graph), and activation was determined in that population showing accumulation using FRET analysis of nuclear regions (data below bar graph; Materials and Methods). (D) Overexpression of YopT blocks translocation of YopE from bacteria into host cells. After 1 h of infection with *Y. pseudotuberculosis* strains at MOI = 50, 0.5 × 10^6^ COS1 cells were extracted with 0.1% NP-40 and separated into soluble and pellet fractions to identify translocated YopE in the soluble fraction (Materials and Methods). One-sixth of the soluble fraction and one-half of the pellet were analyzed by immunoblotting using an anti-YopE antibody. Strains used were as in (A), with the addition of the *yscU* mutant, defective for type III secretion as a negative control.

To directly study the effects of simultaneous deposition of YopT and YopE on Rac1 dynamics without changing translocation rates, two alternate strategies were pursued. First, strains expressing only YopT (“T,” Figure 7) or YopE (“E,” Figure 7) were simultaneously incubated with COS1 cells. Second, Rac1 dynamics were analyzed after incubation of COS1 cells with the recently sequenced clinical isolate IP32953 encoding both YopT and YopE on the *Yersinia* virulence plasmid [32]. Coinfection of the YopE- and YopT-expressing strains resulted in a strong reduction in cytoplasmically localized Rac1 activation relative to a strain lacking YopE (compare “E + T” to “T,” Figure 7A and 7C), although the levels of activation were somewhat higher than that seen for a single infection with the YopE-expressing strain (Figure 7A and 7C). In contrast, the effects of YopE on the host cell could be totally blocked by the presence of YopT, if the strain expressing YopT was allowed to incubate with the cells for 30 min prior to the addition of the YopE-containing strain (Figure 7B and 7C). Therefore, the presence of YopT in the cytoplasm of host cells clearly interfered with the activity of YopE. During conditions of simultaneous infection, however, YopE could efficiently compete with YopT to cause inactivation of Rac1. This result is supported by the behavior of IP32953, which showed efficient inactivation of cytoplasmic pools of Rac1 although the strain encodes YopT (Figure 7A).

**Figure 7:**
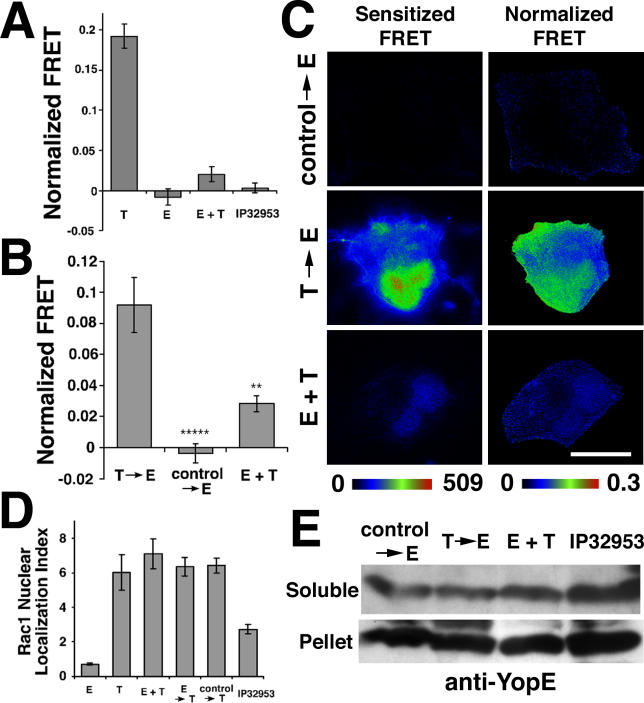
Temporally Regulated Interference of YopE Activity by YopT COS1 cells cotransfected with mCFP-Rac1 and mYFP-PBD were incubated with the noted strains and processed for FRET analysis using ROIs located within the cytoplasm as in Figure 6 (Materials and Methods). (A) YopE can inactivate cytosolic Rac1 in the presence of YopT. FRET was determined after 30 min of infection with COS1 cells for the following strains. E, YP15 *(yopT ^−^yopE ^+^);* IP32953, clinical isolate expressing both YopT and YopE; T, YP17/pYopT2 *(yopT ^+^yopE ^−^);* and E + T, mixed infection of YP17/pYopT2 *(yopT ^+^yopE ^−^)* and YP15 *(yopT ^−^yopE ^+^)* added simultaneously to cells. (B) Injection of YopT prior to YopE interferes with Rac1 inactivation by YopE. Control → E, cells were infected with YP17 for 30 min and then incubated with YP15 (*yopE* ^+^) for 30 min; IP32953, cells were incubated with clinical strain IP32953 *(yopT ^+^yopE ^+^)* for 1 h; and T → E, YP17/pYopT2 *(yopE ^−^)* was introduced onto cells for 30 min prior to incubation of YP15 *(yopE ^+^)* with cells for 30 min more. ***** *p* < 5 × 10^−5^; ** *p* = 0.002. Results from eight cells were quantitated as mean ± SEM. (C) Examples of sensitized FRET and normalized FRET for cells infected with denoted strains, as above. (D) YopT-promoted Rac1 nuclear localization occurs after YopE pretreatment. COS1 cells were incubated with bacteria using the above conditions, with the added protocol of incubating the cells with bacteria expressing YopE for 30 min prior to infection with bacteria that express YopT. E → T, YP15 (*yopE* ^+^ *yopT* ^−^) was introduced onto cells for 30 min, prior to incubation of YP17/pYopT2 (*yopT* ^+^ *yopE* ^−^) with cells for 30 min more. Results are displayed from the analysis of ROIs in eight to ten cells, with measurement of the nuclear localization index for Rac1 (ratio of Rac1 in the nucleus to that in the cytoplasm; Materials and Methods). (E) Translocation of YopE is unaffected by the presence of YopT in either a serogroup I strain or using coinfection conditions. Infection conditions were as indicated above; translocation was assayed, and the amount of protein was determined by immunoblotting using an anti-YopE antibody in an NP-40 extractible fraction (Materials and Methods). Pellet, protein not extracted by NP40 (associated with bacterial pellet); Soluble, NP40-soluble fraction (translocated).

Despite the fact that cytoplasmically-localized Rac1 was inactivated in cells simultaneously exposed to YopT and YopE, YopT still exerted effects on these cells. Nucleus-localized Rac1 could be detected for any experimental procedure that introduced YopT into cells, even if a YopT-expressing strain was added 30 min after the addition of the YopE expressing strain (“E → T,” Figure 7D). This result was not due to lowered YopE translocation, as all infection strategies resulted in efficient translocation of YopE (Figure 7E). It appears that the combined effects of YopE and YopT on a single cell are to inactivate Rac1 in the cytoplasm, and promote translocation of Rac1 into the nucleus.

As the YopT-cleaved Rac1 may be protected from YopE and come in contact with Rac1-specific guanosine exchange factor (RacGEF) proteins in the nucleus [22,30], the activation of Rac1 was measured in the nucleus, normalizing FRET to the concentrations of both Rac1 and PBD in this compartment [33]. This normalization strategy clearly detected activation in this compartment, and it was dependent on both GTP loading of Rac1 (N17, Figure 8A) and binding of Rac1 to PBD (C40, Figure 8A). Similarly, strong activation of Rac1 in the nucleus could be detected if YopT were present, no matter what infection conditions were used (Figure 8B). Most significantly, activated Rac1 was generated in the nucleus by the clinical strain IP32935, which expresses YopT and YopE (Figure 8B and 8C), even though incubation with this strain efficiently inactivated the cytoplasmic pool of Rac1 (see Figure 7A). Therefore, simultaneous deposition of both YopT and YopE acted to generate two pools of Rac1 in the cell: an inactive pool in the cytoplasm and an active pool in the nucleus.

**Figure 8:**
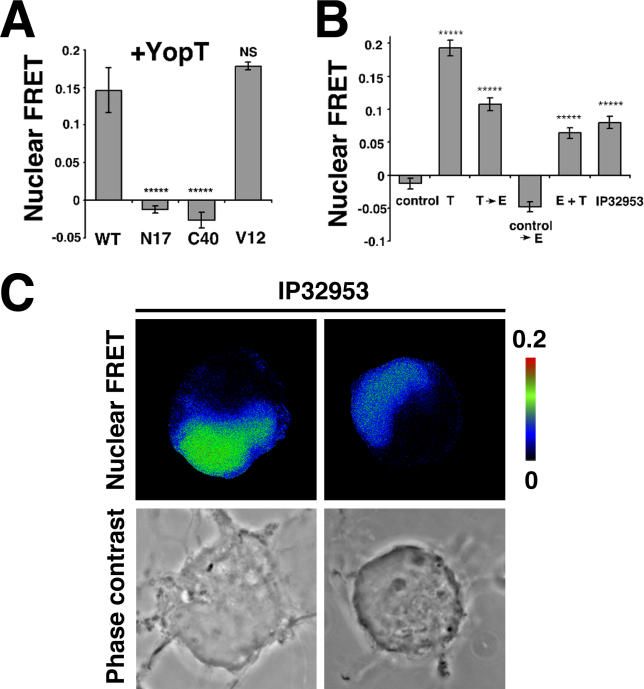
Nuclear Localization of Rac1 Protects It from Inactivation by YopE COS1 cells expressing the probes for Rac1 activity (mCFP-Rac1 and mYFP-PBD) were challenged with the indicated strains and then analyzed for nuclear-localized Rac1 activation using FRET. To account for differences in PBD and Rac1 concentrations in the nucleus relative to the cytoplasm, FRET was normalized to both proteins (Materials and Methods). (A) The FRET readout in the nucleus requires Rac1 activation. Translocation of various mutants of mCFP-Rac1 was promoted by incubating COS1 cells with YP17/pYopT2 for 30 min. (B) YopE is not able to reverse or prevent the accumulation of active nuclear Rac1 promoted by YopT. Control, 30-min incubation with YP17 *(yopE ^−^yopT ^−^);* Control → E, 30-min incubation with YP17 *(yopE ^−^yopT ^−^)* followed by 30-min incubation withYP15 *(yopE ^+^yopT ^−^);* E + T, 30-min coinfection withYP15 *(yopE ^+^yopT ^−^)* and YP17/pYopT2 *(yopT ^+^yopE ^−^);* T, 30-min incubation with YP17/pYopT2 *(yopT ^+^yopE ^−^);* and T → E, 30-min incubation with YP17/pYopT2 *(yopE ^−^)* followed by 30-min incubation withYP15 *(yopE ^+^yopT ^−^)*. ***** *p* < 5 × 10^−5^ relative to control incubation. (C) IP32953 inactivates cytosolic Rac1 and activates nuclear Rac1. Two examples of cells infected with IP32953 for 1 h show intense Rac1 activity (displayed as Nuclear FRET) that corresponds to the nucleus shown in phase contrast images.

## Discussion

Enteropathogenic *Yersinia* sends contradictory signals that control the activation status of Rho GTPases. The most significant result from these studies is that the locale of Rac1 within the host cell was shown to be an important determinant of how the microorganism manipulates GTP loading. Engagement of integrin receptors by the *Y. pseudotuberculosis* invasin protein results in activation of Rac1, but activation was localized around phagosomal cups that harbored *Y. pseudotuberculosis* strains lacking antagonizing Yop proteins (see Figure 2) [10]. Similarly, appropriate localization of Rac1 was required for YopE to effectively block GTPase activation. In contrast, misregulation by YopT was tightly associated with mislocalization of Rac1, with the consequence that the GTPase was activated in the nucleus. Our FRET-based assay revealed rapid inactivation of Rac1 by YopE (see Figure 3) that was surprisingly limited to membrane-associated Rac1. Previous results indicating that YopE localizes to perinuclear vesicles after translocation do not explain why plasma membrane-associated Rac1 should be selectively targeted by YopE [34]. Perhaps YopE is transiently associated with the plasma membrane prior to vesicle-associated retrograde trafficking to a perinuclear compartment, where it encounters other Rho family members [35]. YopT targeting of the membrane-localized Rac1 to the nucleus indicates that there is a novel role for YopT in sending activated proteins into this compartment (see Figure 6). As both effectors targeted the same pool of Rac1, mislocalization of Rac1 by YopT interfered with the ability of YopE to inactivate Rac1, indicating a potential novel role for YopT.

All standard criteria, including the use of Rac1 dominant inhibitory mutants and effector binding-defective derivatives, indicate that our FRET system faithfully monitored the localization of Rac1 activation within the cell and gave activation readouts similar to previously described analysis (see Figure 1) [23]. Our results specifically concerning the mutant-activated Rac1V12 form, however, did differ from a previous report [13]. The relative amount of FRET produced by Rac1V12 at sites having concentrated Rac1 in the membrane was reported to be higher than at other sites in the cell, even when normalized for the increased concentration of Rac1 found at these sites. The contrasting results are almost certainly due to the amount of RhoGDI relative to RacV12 in the two studies, as the binding of RhoGDI to soluble RacV12 interferes with binding to PBD, lowering the FRET readout in cytoplasmic regions expressing relatively high levels of RhoGDI [13]. Consistent with this interpretation, we found that overexpression of RhoGDI greatly attenuated cytoplasmic FRET from mCFP-Rac1V12 to mYFP-PBD (unpublished data). We think it is highly unlikely, however, that our data on localization of mCFP-Rac1(WT) activation were dependent on levels of RhoGDI in our cell line. Activation of mCFP-Rac1(WT) was independent of the concentration of mCFP-Rac1, indicating that even at the lowest levels of Rac1 expression there were enhanced levels of GTP loading at the phagosomal cup compared to other sites in the cell (see Figure 2E).

A signal that induces the release of Rac1 from RhoGDI is the first step in activation after integrin engagement, followed by GTP loading by guanine nucleotide exchange factors and translocation of Rac1 to the membrane [13]. During invasin-promoted uptake, these events could occur sequentially, or be coupled to each other in a concerted process involving a RacGEF. Dock180 is a RacGEF that is a likely candidate for involvement in this process, as it has been implicated in both integrin-dependent activation events and in phagocytosis [36,37]. This protein is recruited to complexes resulting from tyrosine phosphorylation cascades that occur subsequent to integrin engagement [38]. In fact, high efficiency invasin-promoted uptake requires tyrosine phosphorylation, but there is considerable bacterial uptake even in the presence of tyrosine kinase inhibitors [39]. Therefore, we favor a model in which there are multiple pathways leading to Rac1 activation after bacterial binding, some of which may involve tyrosine kinase cascades.

It has been argued that misregulation of the host cell cytoskeleton by the *Yersinia* translocated effectors is a two-step process that involves Rac1 inactivation followed by removal of the protein from the membrane after integrin engagement. RhoGDI removes Rac1-GDP from the membrane, preventing reactivation and coupling of Rac1 with downstream effectors [35]. This extraction process may be required for bacterial RhoGAP proteins (such as YopE) to mediate wholesale cytoskeletal changes, because cell rounding induced by the RhoGAP homolog ExoS from *P. aeruginosa* is dependent on the presence of RhoGDI [40]. It is possible that most *Yersinia* strains bypass the necessity for using such a host factor to extract YopE-inactivated Rac1, because YopT cleavage results in removal of Rac1 from the membrane, giving the potential for the two virulence factors to work in concert (discussed below). The reason that removal from the membrane is important is not clear, but Rac1-GDP present in the membrane may be transiently reactivated, reversing the effects of the RhoGAP.

Rac1 released from the plasma membrane by YopT resulted in an active pool of the GTPase within the nucleus (see Figures 4 and 8), consistent with the notion that the polybasic C terminus of Rac1 acts as a nuclear localization signal when the prenylation site is removed [22]. In the experiments described here, this proteolysis interfered with the activity of YopE (see Figure 7B). Such interference would appear to be counterproductive, with YopT ruining the effects of YopE on the host cell. However, the behavior of the *Y. pseudotuberculosis* I strain IP32935 clarifies this relationship, as the competition between YopT and YopE is not an all-or-none process. During incubations with this strain, a pool of activated and nucleus-localized Rac1 was generated while the cytoplasmic pool of Rac1 was inactivated. Therefore, the combined action of YopT and YopE generated two spatially distinct pools of Rac1 having different activation states (see Figures 7 and 8), allowing fine-tuning of the activation properties of the GTPase. It is interesting to note that this strategy may be important for only some *Yersinia* species, or for causing disease in only a subset of mammalian hosts. Although YopT is expressed in most of the known *Y. pestis* and *Y. enterocolitica* strains, the situation is less clear-cut with *Y. pseudotuberculosis,* in which clinical isolates such as YPIII lack expression of YopT. For these strains, or for the hosts in which they grow, nuclear localization of Rac1 may not be important, and may even interfere with the disease process. In fact, the presence of YopT was observed to reduce virulence of *Y. enterocolitica* in the mouse [41]. This result is presumably not true for all hosts, as there must be some selection for retention of this translocated protein. Alternatively, YopT may be important for a process other than acute disease, such as maintaining a persistent infection.

Although competition between YopT and YopE can be observed in this work, it is possible that the two proteins have a collaborative relationship that is more difficult to measure. YopT could collaborate with YopE by releasing Rac1-GDP from the membrane after YopE action but prior to that of RhoGDI, causing accumulation of inactive Rac1 in the nucleus. This should bypass any need for RhoGDI to support the function of YopE as previously reported [40]. The activation state of Rac1 in the nucleus could then be controlled by GEFs in this compartment.

As a final note, the translocation of Rac1 into the nucleus that results from YopT cleavage may be a strategy to cause misregulation of the host cell transcription program. As the nuclear Rac1 population includes active protein, it is able to bind downstream effectors. Furthermore, it is known that several GEFs, such as SmgGDS and Dock180, can be similarly localized in this compartment, maintaining Rac1 in the active conformation to bind nuclear factors [22,30]. One such factor that interacts with Rac1-GTP is STAT3 (signal transducer and activator of transcription 3) [42]. This transcription factor controls interleukin-10-dependent suppression of macrophage activation, so YopT-induced translocation of activated Rac1 into the nucleus may prevent macrophage activation, even in the absence of an external interleukin-10 signal [43]. The ability to measure the activation levels of Rho family members in response to bacterial binding to host cells, and determine the effects of misregulation on inflammatory responses, should facilitate future studies on the potentially competitive roles of *Yersinia* translocated effector proteins.

## Materials and Methods

### Cell culture, DNA constructs, and transfections.

COS1 cells were cultured and transfected as previously described [10]. Plasmids pmECFP-N1 and pmEYFP-N1 containing the L211K mutations in CFP and YFP, which prevent dimerization and reduce the probability of false-positive FRET, were generated by QuikChange site-directed mutagenesis (Stratagene, La Jolla, California, United States) using pECFP-C1 and pEYFP-C1 (Clontech, Palo Alto, California, United States) as templates [26]. T7 epitope-tagged pCGT-Rac1, pCGT-Rac1V12, and pCGT-Rac1N17 were described [14]. The human Rac1 cDNAs were isolated from these plasmids and inserted directly downstream from the 3′ end of CFP located in pmECFP to create pmCFP-Rac1(WT), pmCFP-Rac1V12, and pmCFP-Rac1N17 (see Table 1 for summary of derivatives). The Rac1 Y40C, R66A, or C189S mutations were introduced into pmCFP-Rac1(WT) or pmCFP-Rac1V12 by site-directed mutagenesis using the QuikChange protocol. The pmYFP-PBD and pmYFP-PBD(LL) plasmids were generated by PCR amplifying the region encompassing residues 67–118 of human PAK1 cDNA from pCMV6M-Pak1 and pCMV6M-Pak1(LL) (H83L, H86L), kindly by provided by J. Chernoff (Fox Chase Cancer Center, Philadelphia, Pennsylvania, United States), and then fusing the product to the 3′ end of YFP of pmEYFP. All plasmids were verified by DNA sequencing. The mammalian expression vectors that express N-terminal myc-tagged RhoA and myc-tagged Cdc42 were provided by K. Wong (University of California at San Francisco, San Francisco, California, United States) and Rac16Q by U. Knaus (Scripps, San Diego, California, United States). The bacterial expression vector pYopE was described previously [14]. pYopT, which encodes *Y. pseudotuberculosis* serogroup I YopT under the strong YopH promoter, was a kind gift from J. Bliska (The State University of New York, Stony Brook, New York, United States). *Y. pseudotuberculosis* pYPIII(P^−^) is a serogroup III strain cured of the virulence plasmid and that lacks the YadA protein. *Y. pseudotuberculosis* strain YP17 (YPIII[pYV ^+^] *yopT* ^−^ *yopE*::*kan yopH*::*cam*), lacks three translocated effectors but has an intact type III secretion system. *Y. pseudotuberculosis* strain YP15 (YPIII[pYV ^+^] *yopT* ^−^ *yopH*::*cam*) is a *yopE* ^+^ derivative of YP17. The strain IP32935, a kind gift from E. Garcia (Lawrence Livermore National Laboratory, Berkeley, California, United States), is a serogroup I clinical isolate that has recently been sequenced and encodes both YopT and YopE on its virulence plasmid [32].

### *Y. pseudotuberculosis* infection of mammalian cells and immunofluorescence staining of bacteria.

Growth conditions of YPIII(P^−^) were used such that invasin was the only protein expressed that promoted attachment to host cells. Bacteria were grown on LB-agar plates, supplemented with 100 μg/ml ampicillin when necessary, at 26 °C for 2 d. The day before infection, a single colony was grown with aeration at 26 °C overnight in LB broth containing 100 μg/ml ampicillin as necessary, and then subcultured in broth with aeration until A_600_ = 0.7. For YP15 and YP17 strains, subcultures of the overnight cultures were performed in broth supplemented with 2.5 mM CaCl_2_ and grown at 26 °C until A_600_ = 0.2. At this point, the cultures were then shifted to 37 °C and aerated for 1 h. A multiplicity of infection (MOI) = 50:1 was used for YPIII(P^−^) incubations, and an MOI of 25:1 was used for YP17 derivatives. For YP17/pYopE, 0.1 mM isopropyl-β-D-thiogalactopyranoside was supplemented during infection to induce YopE expression.

COS1 cells incubated with bacteria were processed for immunofluorescence staining using anti-*Y. pseudotuberculosis* as described [10], revealing extracellular bacteria with anti-rabbit IgG-Texas Red (Molecular Probes, Eugene, Oregon, United States) and total bacteria with goat anti-rabbit IgG-Cascade Blue (Molecular Probes) after fixation with 3% paraformaldehyde.

To detect localization of Rac1, Cdc42, or Arf6-HA, cells were stained using mouse monoclonal antibodies directed against Rac1, Cdc42 (BD Transduction Laboratories, San Diego, California, United States), or the HA tag (Santa Cruz Biotechnology, Santa Cruz, California, United States) followed by probing with goat anti-mouse IgG-Alexa Fluor 488 (Molecular Probes). To analyze partially engulfed organisms, bacteria were identified in which only a portion of the rod showed staining with anti-*Y. pseudotuberculosis* in the absence of permeabilization [14]. To determine the extent of Rac1 accumulation in the nucleus, images were captured from single cells, and the mean intensity values of the nucleus as well as that of the cytoplasm were determined. The Rac1 nuclear localization index was then defined as the ratio of the two intensity values (mean nuclear intensity divided by mean cytoplasmic intensity) from eight to ten cells.

### FRET image analysis to determine levels of Rac1 activation.

To determine levels of Rac1 activation within COS1 cells, monolayers grown on 12-mm untreated round glass coverslips (Fisher Scientific, Pittsburgh, Pennsylvania, United States) were washed three times in PBS, fixed in PBS containing 3% paraformaldehyde, and then immunostained, if appropriate. The coverslips were then mounted on glass slides in the presence of antifade reagent (BioRad, Hercules, California, United States). Individual cells were analyzed using a Plan Apo 100×/1.4 objective fitted on a Nikon Eclipse TE300 inverted microscope (Nikon, Tokyo, Japan) with a 100-W Hg light source, using neutral density filter-4 (25% transmittance). Images were captured using a Hamamatsu Orca II camera (Hamamatsu Photonics, Hamamatsu City, Japan), with scripts controlled by IPLab v 3.5 software (Scanalytics, Rockville, Maryland, United States) capturing, in order, the YFP, CFP, and FRET channels at a bin setting of 2 × 2 with 200 msec exposure time. Filter sets (Chroma, Rockingham, Vermont, United States) consisted of: YFP excitation/emission set with HQ500/20× (exciter), Q515LP (beamsplitter), and HQ535/30m (emitter) filters; CFP excitation/emission set, with D436/10×, 455DCLP, and D480/40m filters; and a FRET set, with D436/10×, 455DCLP, and HQ535/30m filters. Photobleaching of YFP was performed using the D535/50 excitation filter and a Q515LP beamsplitter (Chroma).

The ROI chosen to determine FRET was dependent on whether or not transfectants were incubated with bacteria. For all experiments in which transfectants were incubated in the absence of bacteria, two random fields (each about 10% of the area of a whole cell) located within cytoplasmic regions were chosen. Nucleus-localized protein was avoided, unless stated otherwise. To determine the amount of FRET surrounding nascent phagosomes, the ROI chosen was around partially internalized bacteria [14]. In cases in which the amount of normalized FRET in the area proximal to the phagosomes was compared to elsewhere in the cytoplasm of the same cell, a single ROI of the COS1 cytoplasm, which was near the nascent phagosome and encompassed a similar-sized area to that of the nascent phagosome, was chosen as the normalized FRET control.

To quantitate sensitized FRET, correction coefficients were derived to account for bleedthrough of CFP emission and cross-excitation of YFP during FRET measurements. The background level of fluorescence on the coverslip was determined, and then cells that were singly transfected with either mCFP-Rac1 or mYFP-PBD were analyzed. The correction coefficient for a particular fluor was determined by capturing images from the singly transfected cells. The mean intensities (I) from two randomly-chosen cytoplasmic regions of interests from single cells were determined, and the fraction of emission observed with the FRET filter relative to the appropriate excitation/emission filter set (I_FRET filter_/I_fluor filter_) was calculated. The coefficient of CFP bleedthrough emission into the FRET emission filter (I_FRET filter_/I_CFP filter_) was approximately 0.39, and the coefficient of YFP cross-excitation by the FRET excitation filter (I_FRET filter_/I_YFP filter_) was 0.22. For a sample, sensitized FRET was typically calculated as I_sensitized FRET_ = I_FRET_ − (0.39 × I_CFP_) − (0.22 × I_YFP_) [33]. In certain cases, normalized FRET was determined, which was the ratio of FRET relative to the concentration of mCFP-Rac1 at individual sites within the cell. Images of normalized Rac1 activation were obtained by first constructing sensitized FRET images and masking them, so that all of the non-cell pixels had zero FRET intensity. Then the resulting images were divided by those obtained using the CFP filter. To account for the dramatic differences in the relative concentration of PBD and Rac1 in the nucleus after YopT treatment, we normalized FRET signal from nucleus to both proteins by calculating nuclear FRET as I_sensitized FRET_/(I_CFP_ × I_YFP_)^1/2^ as described previously [33].

Photobleaching was determined after grabbing the images required for FRET determinations. The sample was the exposed for 3 min to a 100-W Hg lamp using a 535 nm excitation filter but no neutral density filter. An image was then grabbed using the CFP filter set. Images obtained before and after photobleaching were background subtracted, and the CFP fluorescence in two ROIs from each image were obtained to calculate the percentage increase in CFP emission.

### Detergent fractionation of COS1 cells infected with *Y. pseudotuberculosis* for Yop translocation.

Freshly diluted *Y. pseudotuberculosis* was grown in LB broth with antibiotic if necessary, as above, at 26 °C for 2 h, transferred to 37 °C, and grown for a further 2 h. The bacteria were then incubated at MOI = 50 for 0.5 or 1 h with COS1 cells (0.5 × 10^6^) grown in 10-cm^2^ dishes. After one wash with PBS, infected cells were incubated for 20 min at 4 °C with 400 μl of lysis buffer (150 mM NaCl, 0.1% NP-40, and 10 mM NaH_2_PO_4_) containing 200 μM AEBSF, 10 μg/ml leupeptin, and 10 μg/ml pepstatin. Lysed cells were collected and centrifuged for 10 min at 13,000 rpm at 4 °C, and 133 μl of the supernatant was taken and precipitated with 1:4 methanol-chloroform. The precipitated detergent-soluble proteins and the detergent-insoluble pellets were resuspended and boiled in SDS-PAGE sample buffer containing 5% SDS. Half of the soluble and pellet samples were analyzed by SDS-PAGE and immunoblotted using an antibody against YopE, provided by J. Mecsas (Tufts University, Massachusetts, United States).

## Abbreviations

FRET: fluorescence resonance energy transfer
GTPase: guanosine triphosphatase
I: intensity
MOI: multiplicity of infection
PBD: p21-binding domain
RhoGAP: GTPase-activating protein for Rho family members
RhoGDI: Rho GDP-dissociation inhibitor
RacGEF: Rac1-specific guanosine exchange factor
ROI: region of interest
SEM: standard error of the mean
WT: wild-type
